# FAIR Digital Twins for Data-Intensive Research

**DOI:** 10.3389/fdata.2022.883341

**Published:** 2022-05-11

**Authors:** Erik Schultes, Marco Roos, Luiz Olavo Bonino da Silva Santos, Giancarlo Guizzardi, Jildau Bouwman, Thomas Hankemeier, Arie Baak, Barend Mons

**Affiliations:** ^1^Leiden Institute for FAIR and Equitable Science, Leiden, Netherlands; ^2^GO FAIR Foundation, Leiden, Netherlands; ^3^Human Genetics Department, Leiden University Medical Center, Leiden, Netherlands; ^4^Faculty of Electrical Engineering, Mathematics and Computer Science, University of Twente, Enschede, Netherlands; ^5^Faculty of Computer Science, Free University of Bozen-Bolzano, Bolzano, Italy; ^6^Netherlands Organisation for Applied Scientific Research, Zeist, Netherlands; ^7^Leiden Academic Centre for Drug Research, Leiden University, Leiden, Netherlands; ^8^Euretos, Utrecht, Netherlands

**Keywords:** nanopublications, data stewardship, FAIR guiding principles, machine learning, FAIR Digital Twin, FAIR Digital Object, Knowlet, augmented reasoning

## Abstract

Although all the technical components supporting fully orchestrated Digital Twins (DT) currently exist, what remains missing is a conceptual clarification and analysis of a more generalized concept of a DT that is made FAIR, that is, universally machine actionable. This methodological overview is a first step toward this clarification. We present a review of previously developed semantic artifacts and how they may be used to compose a higher-order data model referred to here as a FAIR Digital Twin (FDT). We propose an architectural design to compose, store and reuse FDTs supporting data intensive research, with emphasis on privacy by design and their use in GDPR compliant open science.

## Introduction

The promise of meaningful and explainable “Artificial Intelligence” in research and innovation can only be fulfilled when as much data as possible is made FAIR, both in the original sense of automating operations that support find ability, accessibility, interoperability, and reusability (Wilkinson et al., 2016), but also in the derivative sense of data and services that are Fully AI Ready. Moreover, any computable agent, such as virtual machines visiting FAIR data repositories, should themselves be FAIR and FAIR-enabled, i.e., they should themselves follow the FAIR principles (being FAIR) and they should be able to consume FAIR data and interact with other FAIR computable agents (being FAIR-enabled). This means that both data and the associated computing services need to be constructed as FAIR Digital Objects (FDOs) and adorned with sufficiently rich metadata to operate as independently from human intervention as possible (Collins et al., 2018). Although the technical specifications of FDOs are still undergoing revision^1^, we borrow heavily from the FDO approach to introduce the concept of an FDT through the layered extension of the digital twin definition:

- *The Digital Twin (DT) of a physical object in the real world is a data model accommodating the comprehensive collection of digital information available about that physical object*.
- *A FAIR Digital Twin (FDT) is when the collection of digital information composing the twin is FAIR and thus machine actionable*.
- *The FAIRness of the FDT is guaranteed when all components of the FDT, and hence the FDT as a whole, are represented using FAIR Digital Objects (FDOs) and thus adhere to both the FAIR Guiding principles and the FDO specifications*.

It follows that not every FDO is necessarily a FDT, although a proper FDT would be built entirely from more fundamental FDOs, in a modular and recursive fashion. By composing FDTs as collections of FDOs, it can be guaranteed that the FDT itself fulfills the requirements of an FDO. Furthermore, in the FDT definition, we expand the range of possible referents of the twin to include not only “real world objects” such as a person, an organ, an instrument, or a blood sample but also of “mental constructs” which are concepts (types) without an identifiable physical instance (a physical twin), such as the concepts “cancer,” “trust,” or “humor” (Guizzardi and Porello, 2019).

By ensuring that FDTs are themselves examples of FDOs, any FAIR-enabled computational agent should be able to automatically interoperate with FDTs as passive, although FAIR-ready, research objects. This would mean that collections of FDTs could be visited or shared under well-defined conditions and analyzed with user-defined methods. However, FDTs could also inform active research agents that roam the internet of FAIR data and services to, for example, search for similar FDTs (“find patients like me” or “find tumors like me”). Beginning with the definition above, we develop here some of the design principles and a high-level conceptual model of a FDT, how such FDTs can be constructed in a modular fashion, and a few visionary examples of how FDTs can be used in scientific communication, promoting data repurposing and scientific reproducibility.

## A Fair Digital Twin for Scientific Research

The concept of a digital twin originated in 2002 (Grieves, 2019) from the engineering and product manufacturing field where the DT of, for example, a jet engine is a real-time digital representation of a particular physical jet engine identifiable by its unique production serial number. The digital twin serves as a platform for monitoring the performance of that particular physical object over its life cycle, in testing simulations and in predictive maintenance. The DT can track not only the performance of the product, but also other kinds of information such as administrative details like maintenance logs and down-time, technicians who service the product, and associated maintenance and running costs.

However, for FDTs to be of use for data-intensive open science, they need also to support the processes driving knowledge discovery, including the computationally assisted navigation of knowledge graphs built from tacit associations and semantically latent (and often novel) relationships. FDTs must also autonomously manage what are considered difficult semantic problems associated with “near-sameness” and “conceptual drift.” Hence, we propose here that we can, and indeed we are compelled, to extend the idea of FDT beyond instances of physical objects to also mental constructs that are fundamental to knowledge discovery processes. To do this, we draw on the ontological distinction between types and instances, i.e., cancer as an abstract *type* that captures a pattern of regularities observed across multiple individual physical instances of tumors.

The primary purpose of the FDT is to be a digitally enabled container of the collective knowledge about a given subject, in this case, the general concept of “cancer” as it is known today by science. Moreover, cancer only manifests itself in the physical world in individual tumors that on the one hand have only a subset of properties of the general concept type “cancer” but on the other hand have additional properties like occurrence in a specific tissue, a particular person, in a particular time frame, and undergoing a particular treatment regimen. When extending the concept of FDT to include types, it is important to clarify that there are regular patterns that persist and maintain the identity of the concept even as our knowledge progresses. As we learn more about cancer, the concept qualitatively undergoes changes (as reflected by the nature of its changing associations to other concepts) while essentially retaining the identity of the same underlying general phenomenon (i.e., the type or, what philosophers call the same universal). The inevitable “conceptual drift” that emerges with each new data point or expert commentary, is something we need to capture in the digital world to make machines better equipped to serve our sophisticated knowledge discovery needs. Here we use the term “concept” [defined in Mons and Velterop (2009) a *unit of thought*] to refer to both “types” and “instances” of types.

We therefore allow the conceptual model composed of a collection of properties related to a concept which is the referent or “subject” of the FDT. In the FDT, this collection of properties is populated by machine-actionable and -interpretable data elements about the subject (Mons and Velterop, 2009), with sufficiently rich and separate, but perma-linked FAIR, machine-readable metadata. By virtue of being an FDO itself, a FDT includes descriptions allowing machines to detect and resolve the FDT via its globally unique identifier to metadata that (i) make the best possible interpretation of what type the object is, (ii) what operations on it are technically possible, and (iii) what is allowed to be done with the individual FDOs contained in the FDT. It follows that not every digital twin is necessarily FAIR, and not all FAIR data or machine-readable files qualify as an FDT.

The actual subject of a FDT can refer to anything, ranging from a type or instance of molecules to containers of data and executable workflows, to any one of the 3 billion biological specimens in European natural history collections, to citizens in the FAIR-driven research and care environments such as the developing Health-RI research infrastructure in The Netherlands^2^ or the European Open Science Cloud^3^. To emphasize a key aspect again; FDTs (even when very complex) are intrinsically modular and composed of a collection of smaller FDOs, which can be among entities, semantic assertions related to the FDTs subject.

Finally, we also introduce the concept of the *qua* of a FDT, the Latin term meaning “in the character of” (Masolo et al., 2005; Guizzardi, 2006). For example, consider a personal FDT i.e., a digital representation of people throughout their life journey. Typically, a personal FDT will be composed of other FDTs (also FDOs), for instance an FDT of a particular internal organ, a disease, the collection of genomic variants diverting from a reference genome, or sequential metabolic profiles. We refer to these as component FDTs. The “component” status of FDTs is relative, for any given FDT in one case, could be a component FDT in another. For the answer to any research question, only a subset of the FDTs will likely be needed. Therefore, selected subsets of the component FDTs can be “exported” from the personal FDT for given research purposes, avoiding the need to expose all information in every case. This ability to “filter” the FDT for relevant component FDTs enables customized “point of view” on a person ‘*qua'* COVID-19 patient or ‘*qua'* subject of clinical trial. It goes without saying that citizens should be enabled to control who can see which elements of their FDT and for what purposes. Moreover, individuals should also be able to exploit their FDT for “citizen science” as they see fit, whether it be interesting information pertaining to their health risks, or their exposure to toxic chemicals in their neighborhoods^4^. *Qua* filtering on personal FDTs will also help to minimize the risk of unauthorized re-identification of the actual person represented by the FDT.

## Fair Digital Twin as Versatile, Lightweight Scientific Models

We believe that the concept of a FDT as it is defined here, is a natural and straightforward generalization of the original concept of “digital twin,” as it is derived from the engineering and manufacturing domain where a physical object is represented by a mathematical model that replicates some aspect of the physical object's behavior. Any computed feature of a physical object (via a mathematical model or computer simulation) can itself be treated also as an assertional relation. For e.g., object has computed feature. The FDT simply generalizes this originating construct to include assertions of other types (i.e., types other than “mathematical model”). Restricting the definition to “mathematical model” only, preempts other kinds of assertional relations (data/metadata acquired about the object, statements made by people, etc). In this regard, the definition we propose subsumes the original concept of “Digital Twin” as a special case. Furthermore, we believe that no observation, and indeed, no assertion, comes “model free.” Even an assertion as innocuous as a date/time stamp (i.e., ordinary metadata) carries with it (albeit implicitly) a model of time and a framework for describing time and quantitative time keeping. Again, in principle not fundamentally different than what we might think of as a “mathematical model”.

To comprehend better the differences and potential advantages of FDTs, it can be useful to contrast the FDT concept with an earlier concept of a “virtual physiological human” (VPH) (Hunter et al., 2010). In its ultimate integrated form, a VPH instance could be viewed as a massive systems biology model of a reference *Homo sapiens*. In contrast, the FDT of a particular person (or any of its component FDTs, for example, an FDT of a tumor instance), will be much less complex than a universal, dynamic computer simulation of a reference Homo sapiens. Smaller, fully machine actionable units of information will be more modular and easier to adapt at the level of the individual (for instance differences in immune response). In any case, a FDT could seamlessly communicate with any of the more complex mechanistic computer simulations of tissues, organs, or other parts of a VPH-type knowledge base, as long as these larger data and workflow resources also comply with FDO specifications.

However, even in the era of personalized medicine, where individual variations become the focus, a “reference” model for generic human physiology may still serve a valuable purpose in the same way as a “reference genome” is needed in genomics, as a single arbitrary reference that makes the much larger corpus of observed “variants” easier to describe and store as deviations from the reference genome. Reference models of *Homo sapiens* anatomy or physiology can in a similar way reduce the information stored in any personal FDTs. As such, generic reference models and the FDTs of a particular person (or any other real-world entity for that matter) is thus in fact a machine actionable collection of “everything we explicitly know -or have asserted- about that subject.”

As FDTs data models are composed of multiple assertions to the same subject, and as the required storage space for each assertion is on the order of a few kilobytes, the digital footprint of any FDT is relatively minimal. An assertion is composed by its subject (in this case, the referent of the FDT), a property (sometimes called the predicate) and its value. Each property can be presented by the resolvable identifier of the concept representing this property in a given ontology, while the property value can be either a sequence of characters (e.g., the name of a person or the date of birth) or the resolvable identifier of another digital object (e.g., the concept of Home Sapiens or the image file of a MRI scan). Because of its small footprint (primarily short text strings and identifiers), FDTs can be stored in distributed systems, instantiated (as *quas*) on demand, and when necessary packaged and transferred at minimal costs, even at the level of personal and private use.

## Passive and Active Fair Digital Twins

So far, we have described FDTs mostly as “passive models” or as data representing their physical or intellectual counterparts, but they could become much more, when coupled to FAIR-enabled services. FDT can then be activated as an “intelligent research agent and expert advisor,” becoming for people an “FDT-Avatar.” It “knows” (contains) all machine-actionable data about the person it represents, but it also has access, via the FAIR Orchestration of appropriate external FAIR-enabled data, to all open FAIR compliant virtual machines (VM) as computable templates and workflows. Simple, templated applications, but also automated procedures, could be developed that effectively create *ad hoc* computable agents that use the FDT-Avatar to generate customized services, ranging from simple SPARQL queries to complicated VMs. These could explore the FAIR information space to reveal patterns and test hypotheses, which may in turn lead to reports delivered to the physical twin and trusted third parties. Customized FDT-Avatars could thus become a central technological approach not only for the medical experts treating their patients but for supporting citizen science as well.

The FDT also “knows” all experimental evidence about its “physical twin,” including outcomes of experiments performed on analogous test subjects such as derived organoids, studied in organ-on-a-chip technology, which we may qualify as “experimental twins.” This again means that other FDT's, caregivers or researchers can “communicate” with the FDT (or selected components of it) as an *anonymous proxy to seeing the actual person*. In addition, the person could use user-friendly apps to “instruct” his/her FDT to act on their behalf, as a participant in a virtual clinical trial or as an active virtual researcher having direct access to all FAIRified knowledge in the world and/or as a health/behavioral advisor.

Applications and infrastructure that drive the FAIR Orchestration of FDTs open new possibilities for the equitable access to scientific knowledge as each FDT is in essence equally “intelligent” and unbiased and has access to the same global FAIR knowledge base. Highly complex data visiting exercises will obviously be more expensive but will hardly ever be conducted by single individuals, but rather in the scope of research projects and innovation activities. This approach will thus further support equitable access to information and care as the “intelligence” and “reach” of the FDT is *independent* of the social status of the physical twin.

Again, coming back to new possibilities for identity protection afforded by FDTs, very importantly, an FDT can be “anonymous” to the outside world as component FDTs from it can be decoupled, on demand, from their physical counterparts by removing any information that directly links the FDT to its physical twin (for instance assigning the FDT dynamic pseudonymized unique identifiers). The person can be in charge of that coupling and decoupling service (custom de-identification and re-identification) or entrust this process to qualified third parties.

In that way, FDTs could participate in multiple research projects as anonymous proxies of the person and provide only the information needed for the research in question, minimizing the risk that an unauthorized party can re-identify the person. FDT's can be stored safely in personal vaults and encrypted (whilst some essential metadata are exposed in FAIR format) and key-authorization for decryption can be part of a consent process^5^. This will move the citizen/patient from a position of a more or less *passive data provider* giving informed consent to others to reuse that data, into an *active participant* in the data collection and research process, with optimal access to all other FDTs.

As is known for other anonymous data collections, many aspects of the FDT could potentially enable unauthorized parties to re-identify the person by mining even generic data such as birthplace, age, geolocation. For many research questions, these data may be further “smoothed” to allow meaningful categorization by age groups, larger geographical regions, or disease/risks groups. In addition to FDTs of individuals and of types/concepts, we can also create FDTs of patient collectives or other interest groups. Collectives are technically (plural) individuals. For example, the male population over 20 in the Netherlands is a collective, and so is the population of bats in Wuhan. Logically, collectives are neither types nor sets, and have interesting and well-understood ontological properties (e.g., see Guizzardi, 2011). Associations with the FDTs of patient collectives offer additional options for proxy-based identity protection.

Furthermore, following FDO specifications^6^, metadata about the FDT are separate. Hence, only those subsets of metadata records that are needed to make a particular study useful need ever be shared with the requesting virtual machines, which further decreases the risk for undesired re-identification of the person. Only at the authorization of the physical twin or its trusted third-party proxy, may the exact details of these aspects be revealed or added to a collective and only if they are demonstrably needed to refine the research results or to reveal bias and confounding factors. Reciprocally, generic “advice” can be given to hospitals, for example, for “patients of this type” with the hospital (with a higher consent profile) translating that into personalized advice, so that only citizen-authorized parties will be able to connect that advice to the relevant people.

## How Humans Can Build Fair Digital Twins, and How Computers Can Use Them

The developments around FAIR data and services enabled science are still in their infancy. However, we would argue that all essential technical components and approaches to realize FDTs and their FAIR orchestration, already exist. There will be significant effort needed to connect all the components and design a running, scalable and secure FDT-based research environment within the broader FAIR infrastructure concept, but there are no obvious intellectual or technical blocks, and the technologies described below have already demonstrated their value, albeit in less automated environments. As stated earlier, the most significant barrier to the realization of FDTs, in the opinion of the authors, is thus not technological, but rather, conceptual. Further clarification of foundational concepts is provided below.

How do we move from the traditional research environment where people create queries to be sent to dispersed and non-interoperable databases to a world where we have in essence “one computer” where FAIR services see all FAIR data as their accessible research substrate? These services can then effectively access one global FAIR database (“one database”) (Strawn, 2021), largely independent from the human bottleneck of the non-scalable creation of manual queries or one-off research algorithms by humans and their very limited ability to discover complex patterns in complex data?

**Figure 1:**
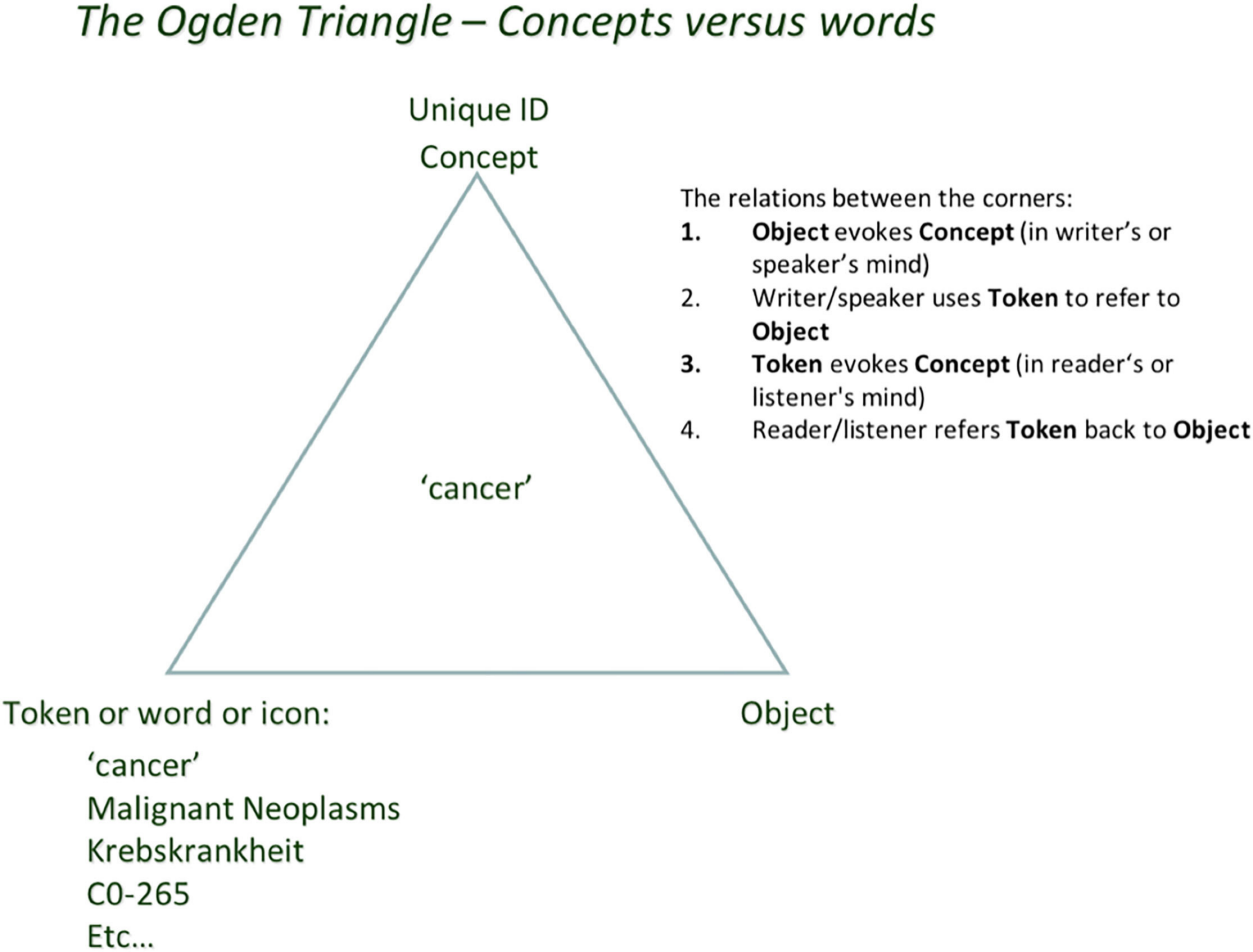
People think in “concepts” as in: “unique units of thought” and how they are meaningfully connected (Mons and Velterop, 2009). That unit of thought can refer to either a physical object in reality, or an “intellectual concept,” an abstraction, or a “mental construct” such as “cancer” (also a “type”) vs. a physical instance of a tumor, or an abstract concept without a physical twin. Humans use a multitude of symbols, words, tokens, and identifiers to “refer to” the concept they have in mind. This ambiguity in human language (and pointedly also in classical scientific narrative) is a source of confusion because of synonyms, homonyms, (mis)translation into other languages, and semantic as well as conceptual drift. All of these will be rather superficially addressed in FDT context later.

The semiotic (or Ogden) triangle (Figure 1) can help us to better understand how the transition from exclusively human (brain) driven science to machine-assisted (and potentially machine-driven) FAIR science can be guided in the near future.

## In Science, We Should Communicate Meaning as Precisely as Possible

Humans can only communicate if we share a semantic domain, so that communicating individuals are mapping from grammatical sentences in a language to that shared semantic domain. In referential semantics, such semantic domains are often referred to as “shared conceptualizations.” Shared conceptualizations are fictions, concepts only really “exist” in people's minds. We align our concepts via shared perception and language. There is always potentially a distance between concepts that are thought to be “shared” by different people. When this distance goes beyond a certain threshold, we create the illusion of “shared conceptualization.” This illusion is broken and potentially dangerous and confusing when we perceive that semantic distance going beyond a given threshold. This threshold is completely context-dependent (the precision I need to talk to a lawyer about a real estate that I am buying is very different from the precision needed to talk to my mother when I tell her the news). When we need more precision, we increase the precision of our language (the language we use in contracts have a much higher precision, ability to discriminate, i.e., a much more sophisticated underlying ontology). This also relates to the subsets of information we wish to share from FDTs for particular purposes. However, the general problem of “near sameness,” is aggravated enormously when machines come into play. Humans deal relatively well with the vague boundaries of the concepts they talk about, and even “false agreement” (in fact two people talk about two -slightly- different concepts, without realizing it) is not much of a problem as long as the “*near sameness*” of the concepts is not jeopardizing the communication of meaning. For instance, if two people discuss a “piano,” it is not always important to distinguish between different kinds of piano's (instruments) as long as they both know they are not talking about “piano” in the sense of the instrument, or in terms of “*sotto voce*,” or they mean “*floor*.” In many cases, the human mind is unconsciously “solving” these ambiguities based on many contextual features (importantly, these are usually not within the narrative itself and thus tacit). Lively human communication (and likely creativeness and humor) *thrives* on subtle differences and vague associations between concepts, and one could even argue this is part of what we assume as “intelligence” and “knowledge discovery by association.” In science, we are taught to convert a scientific conclusion or claim in a language that is as precise as possible, but we collectively violate that rule all the time and we continuously create vague, nested sentences, synonyms, homonyms, naming convention errors and other sources of ambiguity. Now, these in a way “needed” to give a rich human understandable context to our major scientific claims in the narrative, but these texts are a “nightmare for machines,” because machines (so far) deal with precise definitions and notations only. Machines (unless specifically instructed) do not have any access to common-sensical (and, hence, tacit) notions. For example, from “John is married to Mary,” the machine has no way of knowing that “Mary is married to John” (or that “Mary is the wife of John,” that “John is the husband of Mary,” that their life spans overlap etc.). Machines also miss all contextual references and naming conventions that are not explicitly programmed in the digital object environment. Machines see “age” and “Age” as different, unless we instruct them to ignore the capital, but when we use CAPN3 for the human gene and capn3 for the mouse gene, the capitals (which we do all the time) should not be ignored or used in a sloppy way. In this later example, the semantics is not only associated with the term but also with a particular formatting style of the term, which is problematic for both humans and machines if they have not been previously informed about it.

**Figure 2:**
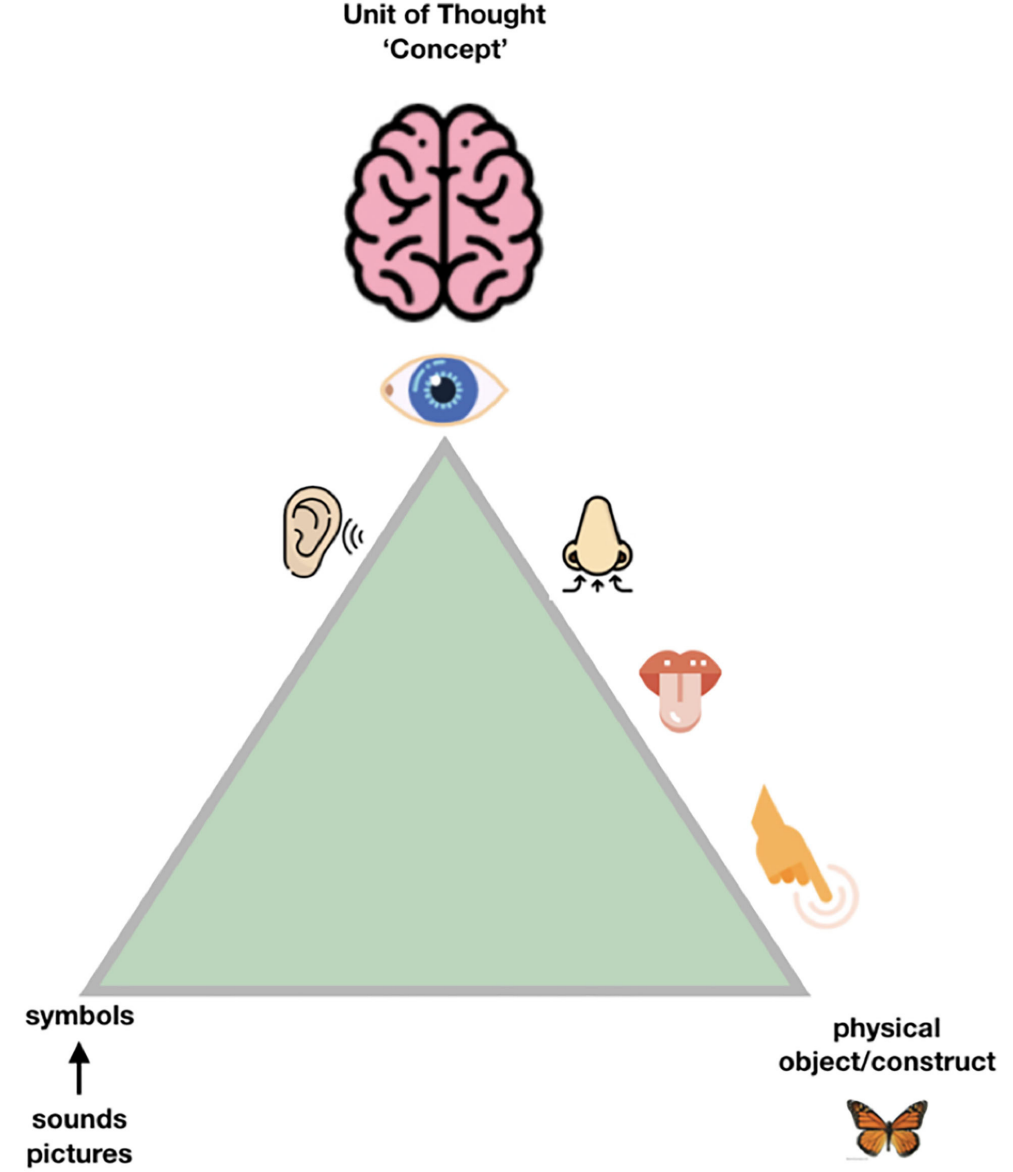
People have multiple “senses” to disambiguate the exact meaning of the object or the contract they are “talking about,” and a unique and highly developed skill set to communicate, even across national languages and jargon, with a reasonable outcome. Still, it could be argued that many misunderstandings between cultures, but even between scientists, can be traced back to either “false agreements” (we think we are talking about the same exact concept, but we are not) and “false disagreements” (we think we talk about significantly different things, while in fact we are not, and the consensus is much bigger than we experience). In science, the sources of ambiguity should ideally be kept to the absolute, unavoidable, minimum. The good news is that moving to machine readable communication of the essence of our scientific findings will also help human communication by reducing both false agreements and false disagreements (Mons et al., 2011).

**Figure 3:**
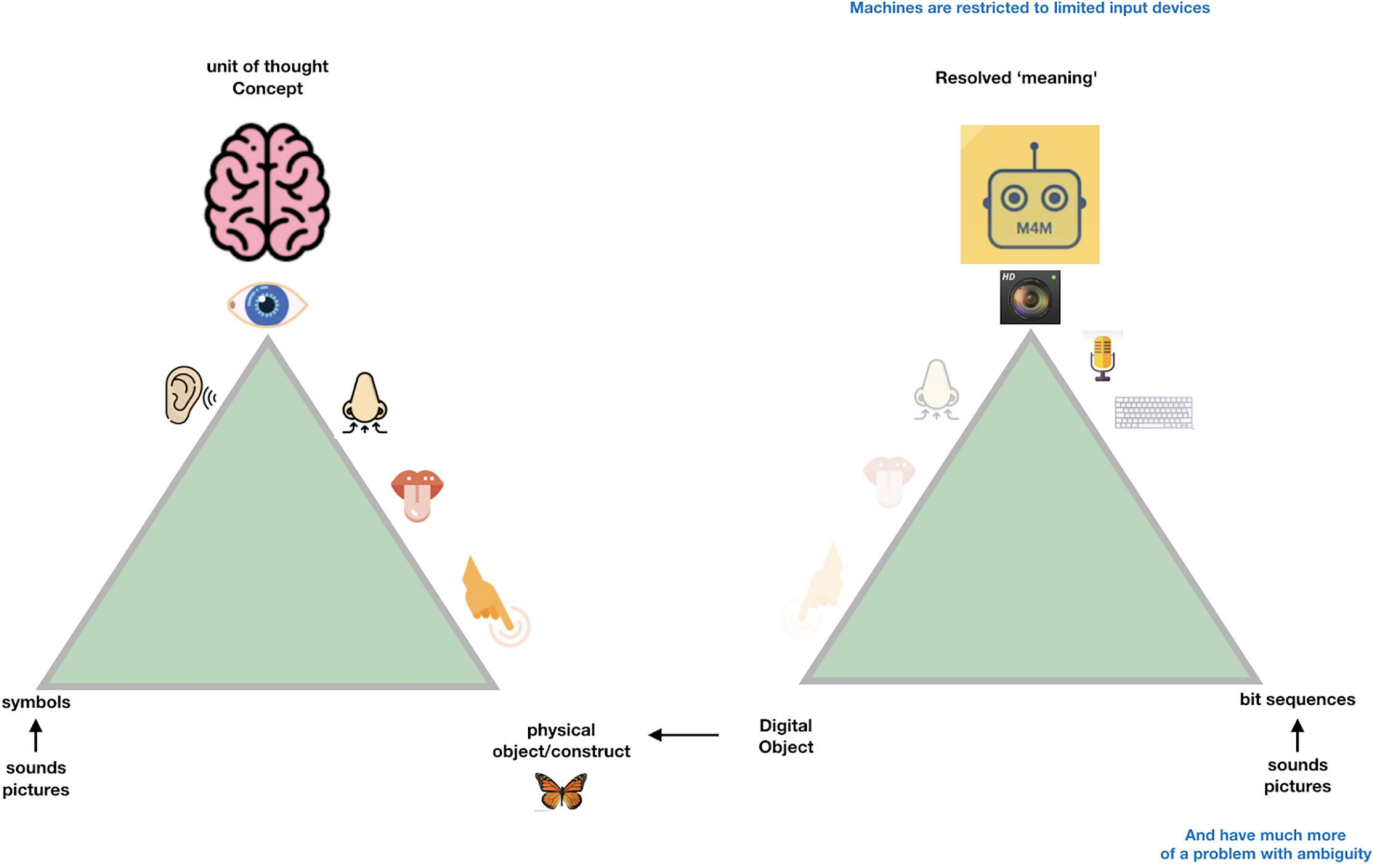
Machines can only deal -internally- with “digital objects.” These are nothing else than a “bitstream” maybe comparable to “series of spikes” in the sensory system of people. While we do not even know whether the same object triggers the same “bitstream” in different people, while they may still call the same emission of light “red,” machines need very precise instructions about the defined meaning referred to by a particular bitstream and therefore each “concept” for a machine (a disease, a drug a person etc.) should have what we call a Globally Unique, Persistent and Resolvable Identifier (GUPRI). In other words, the bitstream that “refers” to a concept (be it another digital object, an object in the physical world, or a mental construct) should resolve (universally) to one, and only one, intended defined meaning at any time. Once we have agreed on such a GUPRI for each concept we deal with in science, we can communicate in a much more precise way, also as humans.

**Figure 4:**
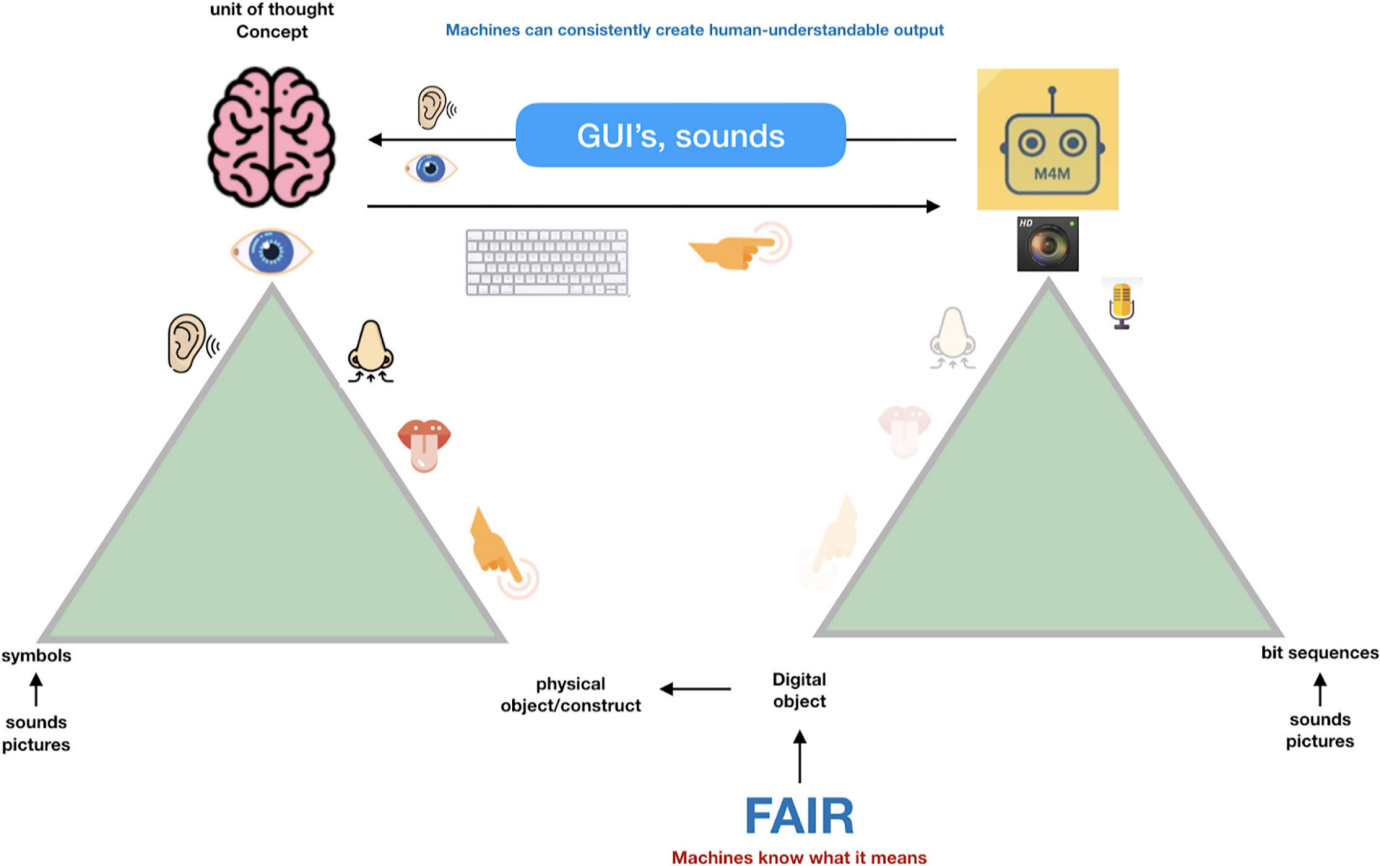
Machines can deal with precise concepts if they are properly defined (the definition preferably also in machine interpretable format). For machines, concepts and their relations should be precisely defined, and it is very important that the association (the “predicate” in semantic triple terms) is also a well-defined concept, with its own GUPRI. Once that is done properly, machines have a magnificent ability to discover and interpret complex patterns in massive amounts of data and information. With simple mapping tables, they are also able to output this information in Graphical User Interfaces and sounds in a human understandable and non-ambiguous way (and in multiple languages).

So, when we try to transition from the “Ogden triangle for people” to the “Ogden triangle for machines,” we need to take the lack of the machine's ability to deal with near sameness, conceptual drift, homonyms and synonyms into account (Figures 2–4).

In Figure 3, we depict the much more restricted connection to the physical world that (for now) machines must deal with. Their essential input is “bitstreams,” regardless of whether these are generated by cameras, microphones, other sensors or keyboards. One could argue that the input to the human brain is also “just” a set of “spikes.” However, the “conceptual picture” that the human mind creates is much more complex than the actual “input” (observation) at that point. In fact, we all have many “associations” (based on an intrinsic conceptual model) with each object we observe, and with each word we hear. This is best to be translated to metaphoric “machine awareness of context” by the concept of a Knowlet described later and in Mons (2019). First, we address the benefit of being “as precise as possible” to respect the limited understanding of machines when we formulate scientific concepts and their relationships (associations, correlations, causal relationships).

The number of concepts we address in research and innovation is rather limited. For instance, in the entire Medline database of >40 million article abstracts, we do not address with any serious frequency more than one million concepts (!) and just over 100 semantic types as defined in the Unified Medical Language System (https://www.nlm.nih.gov/research/umls/index.html). It is the relationships *between* those concepts where the complexity comes in, and we will address this complexity later. First, we need to further define GUPRIs (globally unique and persistent identifiers) beyond what was generically discussed at Figure 3 (Mons, 2019) and in the GO FAIR Foundation criteria document^7^. In this document, principle [F1: (meta)data are assigned globally unique and persistent identifiers] is further specified as being also machine resolvable, and this holds for each concept in the FAIR data realm, not just the “DOI of the entire data set” to mention one frequent misperception about FAIR. Firstly, GUPRIs work as Proper Names or their ontological counterparts: individual concepts. There are multiple sources in the literature regarding the semantics of identifiers. For example, identifiers should obey the following minimal axiomatization: (a) singular reference—they must point to a unique entity, i.e., they are functional; (b) rigid designation—they necessarily refer to the same object (the object can qualitatively change but it is the same object that is changing). It should be emphasized that having two different GUPRIs referring to the same object is perfectly acceptable in machine assisted science (analogous to what is called a “non-unique name assumption” or a synonym) but, because of (b), it means that if two GUPRIs coincide in a world, they coincide necessarily, i.e., they must always refer to the same object. Now, unless we map ontological resources explicitly for such different GUPRIs referring to the same concept, machines will still be unable to determine their “sameness” independently and unambiguously. The Knowlet concept further specified below, will mitigate this issue to a large extent as the conceptual overlap in the Knowlet will begin to enable machines to determine (near) sameness independently from explicit assertions about that sameness (Halpin et al., 2010; Guizzardi, 2015).

Once we have properly defined our concepts and assigned a GUPRI to them, it becomes possible to map all tokens and symbols (including terms in human language) that refer to that concept. First, the machine is now able in a Graphical User Interface (GUI) to depict the custom token of choice. For instance, the GUPRI of the concept “malaria” will yield that word in an English interface for humans, but in a French interface it will yield the word “*paludisme*.” Second, in the Linked Data (i.e., Semantic Web) world, not only explicit links (with defined predicates) between concepts can be made independently from human language, but now also “tacit near sameness” becomes increasingly manageable for machines (see below). Next to enabling machines to assist humans with high throughput and complex data analytics and pattern recognition, it also has a great potential to cross human language (and jargon) barriers, and drive precision in essential scientific claims, which in turn is crucially important for equitable, global science, especially including citizen participation in open science ecosystems as the one proposed here. So, the first step toward FDTs is that we ensure that the “*machine knows what we mean*” always and everywhere on the Internet of FAIR Digital Objects.

Thus, machine readability and interpretability, at the core of the FAIR principles, is needed to make data and information “Fully AI-Ready” (FAIR), ***but humans will benefit along the way***. In fact, a conscious attempt to avoid ambiguity in scientific language will also benefit the quality of narrative in the literature. We will always need “hedging” and rhetoric in scholarly communication, especially also in the social sciences and humanities, but also to nuance certain claims or make them conditional. However, ultimately, science is about precise scientific claims and innovation should be based on solid evidence, not vague associations. It is thus not inconceivable to publish all scientific claims as precisely as possible in “triple format” (Mons et al., 2011). This is in fact the process of performing ontological analysis and fixing the real-world semantics as a prerequisite for defining formal semantics. This process clearly benefits human understanding of concepts! To explain a certain phenomenon requires precisely identifying the nature of that phenomenon. For example, if we know something is an event, we know it has objects as participants, it brings about changes in the world, it unfolds in time, it has a cause (a disposition or set of dispositions), etc. Once we analyze that event, then we can codify/axiomatize all those properties in a way that machines derive from (for instance) a type of ontology and can predictably execute (Guizzardi et al., 2021).

So, we venture to make the claim that Fully AI-Ready really requires precisely defined real-world semantics and not only formal semantics. In a nutshell, ontologies as conceptual tools for analysis and conceptual clarification are fundamental and prior to ontologies as computational logic theories.

**Figure 5:**
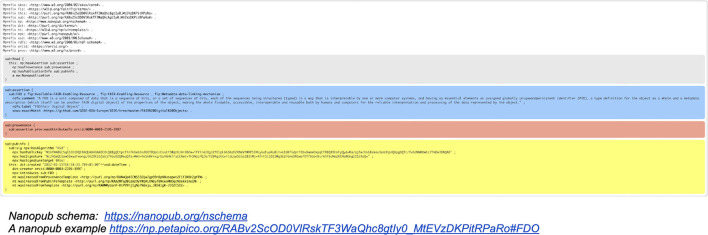
An example of a nanopublication file in RDF format. Please note that the different colored sections, which appear here as one file, are in fact going to be stored in separate, but linked containers as indicated in the current FDO schema. This process is currently ongoing, and the outcome of the process will be reported in a technical paper to be published soon. Online versions of these impressions can be found here: a nanopublication schema^9^ and a nanopublication example^10^.

For more than a decade (Mons, 2005), we have argued that all “precise” scientific assertions (claims) should be published, next to the narrative form, in a machine-readable format. In Mons and Velterop (2009) and Groth et al. (2010) the concept of a “nanopublication” was coined as the smallest meaningful assertion, expressed in RDF^8^ (Figure 5). The minimal “container” of a nanopublication is a single subject-predicate-object (s-p-o) triple of the type [dexamethasone (subject)] [inhibits (predicate)] [prostaglandin E2 (object)], which is a crucial piece of knowledge for COVID-19 treatment for example. Triples are being used extensively throughout the semantic web and in many commercial and industrial applications, but in many cases, the lack of provenance (for instance, who made the claim, based on which evidence -article, data- and when), is lacking or insufficient to allow flawless and provenance rich reasoning with AI applications to use the data. We will also argue later that even proper provenance and truth of individual nanopublications does not fully prevent AI algorithms from going astray.

Nanopublications, however, do have that rich provenance, as later also specified in the FAIR guiding principles (Wilkinson et al., 2016), and, once constructed as proper FDOs (with the metadata separated, but perma-linked to the assertional graph) can therefore fundamentally change scientific and scholarly communications and re-defined research approaches. Incidentally, the established publishing industry has long been remarkably resilient against this relatively simple, but game changing technology, and it is only recently that a GO FAIR implementation network of publishers has formed to enable FAIR compliant publishing, but recent developments are very encouraging (Velterop and Schultes, 2020).

## Stepwise and Modular Building of the Fair Digital Twin

As described in more detail before (Mons, 2019), typical key assertions have been created and re-asserted (preferably properly “cited”) in the literature and on the Web many times, typically around 1,000 times on average, but obviously ranging from one -up to tens of thousands of times for common assertions. Once properly referenced by a GUPRI and constructed as FDOs (in this case of the FDO type < nanopublications>) it becomes trivial for machines to trace and cluster billions of such FDOs in any given analytics procedure. A very simple first step is the clustering of multiple nanopublication graphs that have the exact same content. The result would be a new container (FDO) with exactly the same assertional content as each of the individual, identical nanopublications it was based on, but with a different type: **cardinal assertion** (Gibson et al., 2012).

**Figure 6:**
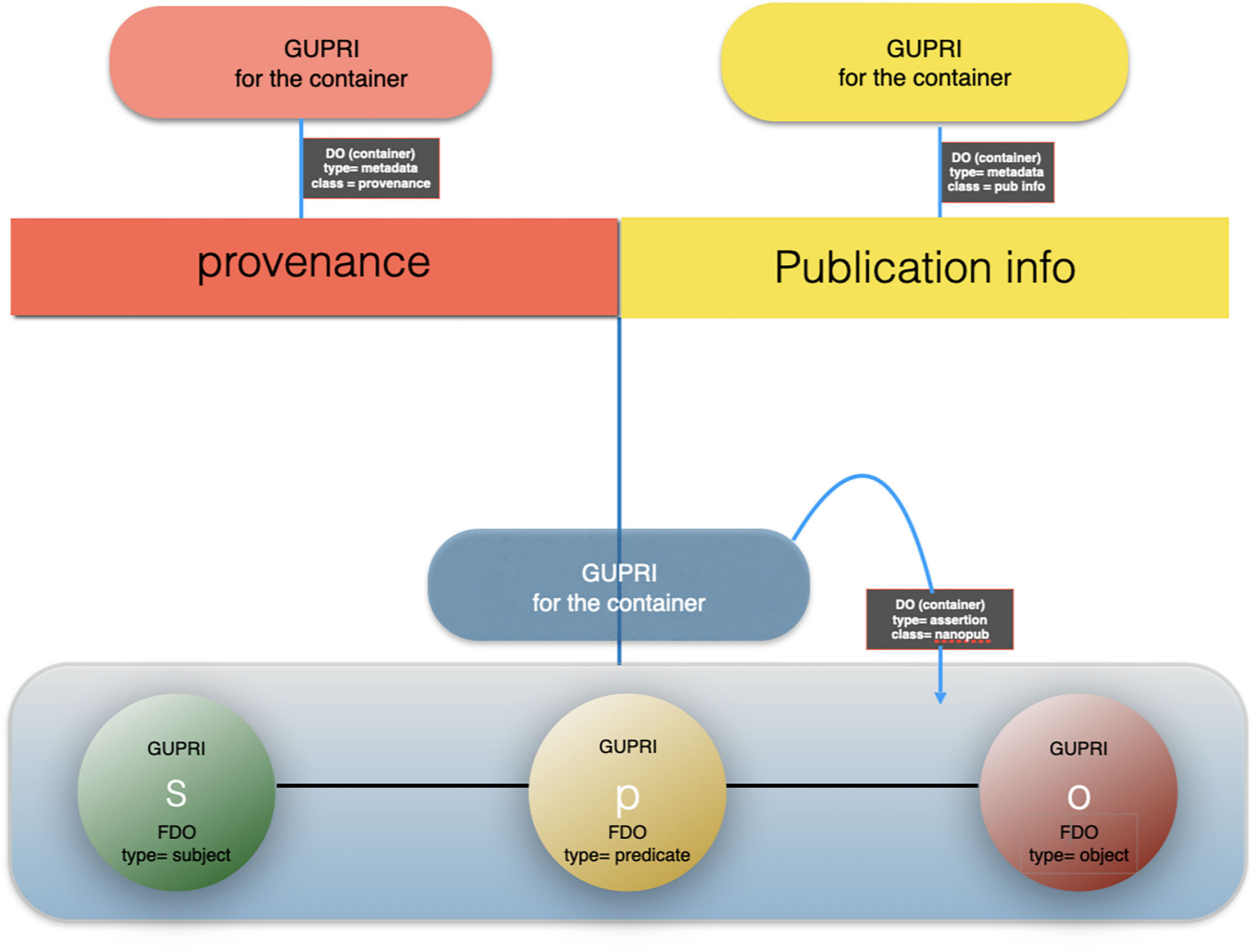
The artist impression of the anatomy of a nanopublication, building on the earlier definitions of Mons and Velterop (2009) and Groth et al. (2010) as a FAIR Digital Object (FDO)^9^: the subject, the predicate and the object are all referred to with a GUPRI (usually in RDF context). The “container” of the triple is typed RDF triple, or when more elaborate, RDF graph and has itself a GUPRI. Its metadata (mostly multiple containers, continuing multiple assertions/triples about the nanopublication) are FDOs in and of themselves, annotated with the proper type and their own GUPRI. Following the FAIR principles, each metadata container contains the explicit reference to the FDO it points to via that FDOs GUPRI. We have argued for a decade now that all precise assertions (i.e., “claims”) in science should preferably have the form of machine actionable nanopublications.

**Figure 7:**
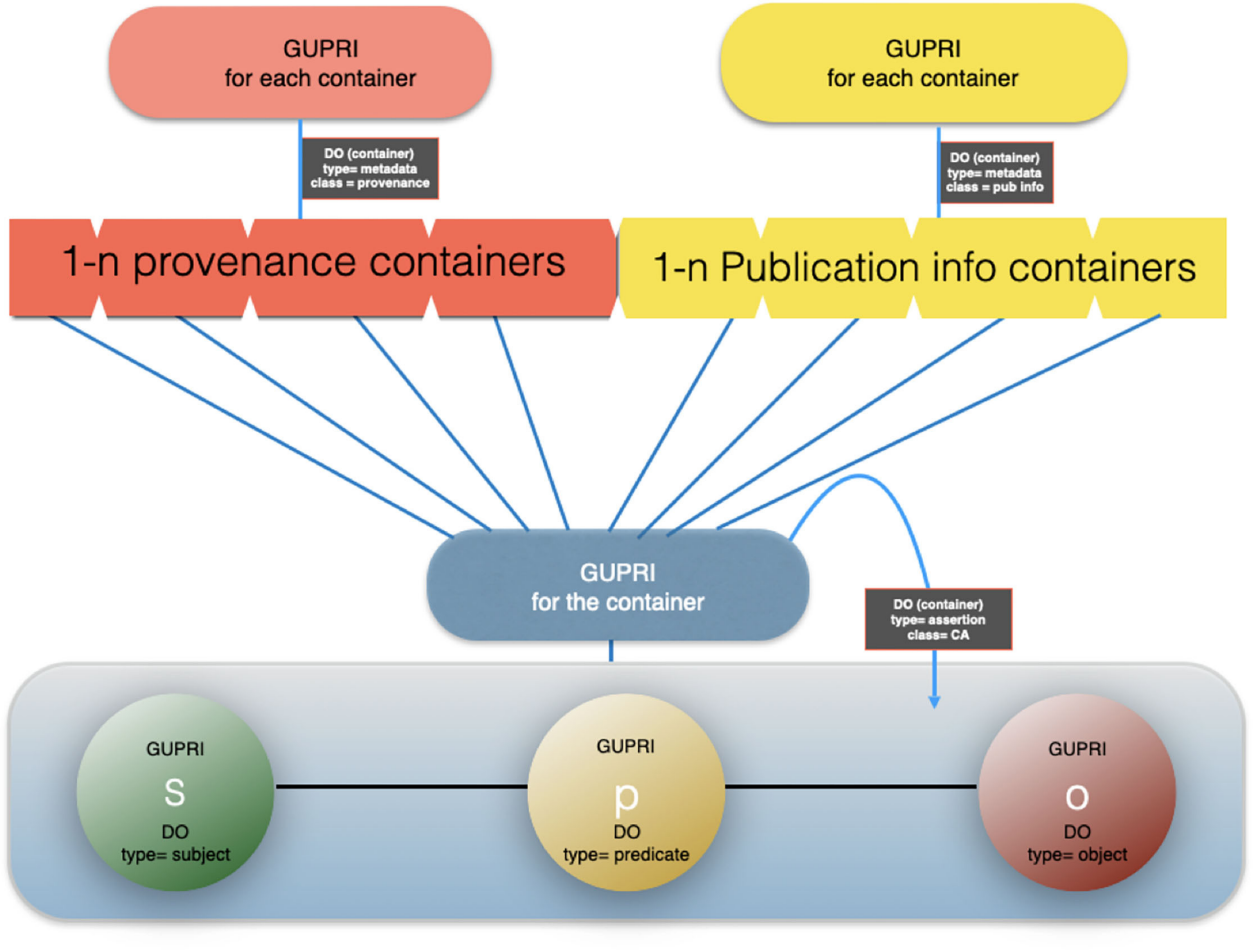
An artist impression of a cardinal assertion with multiple provenance and publication metadata files associated.

**Figure 8:**
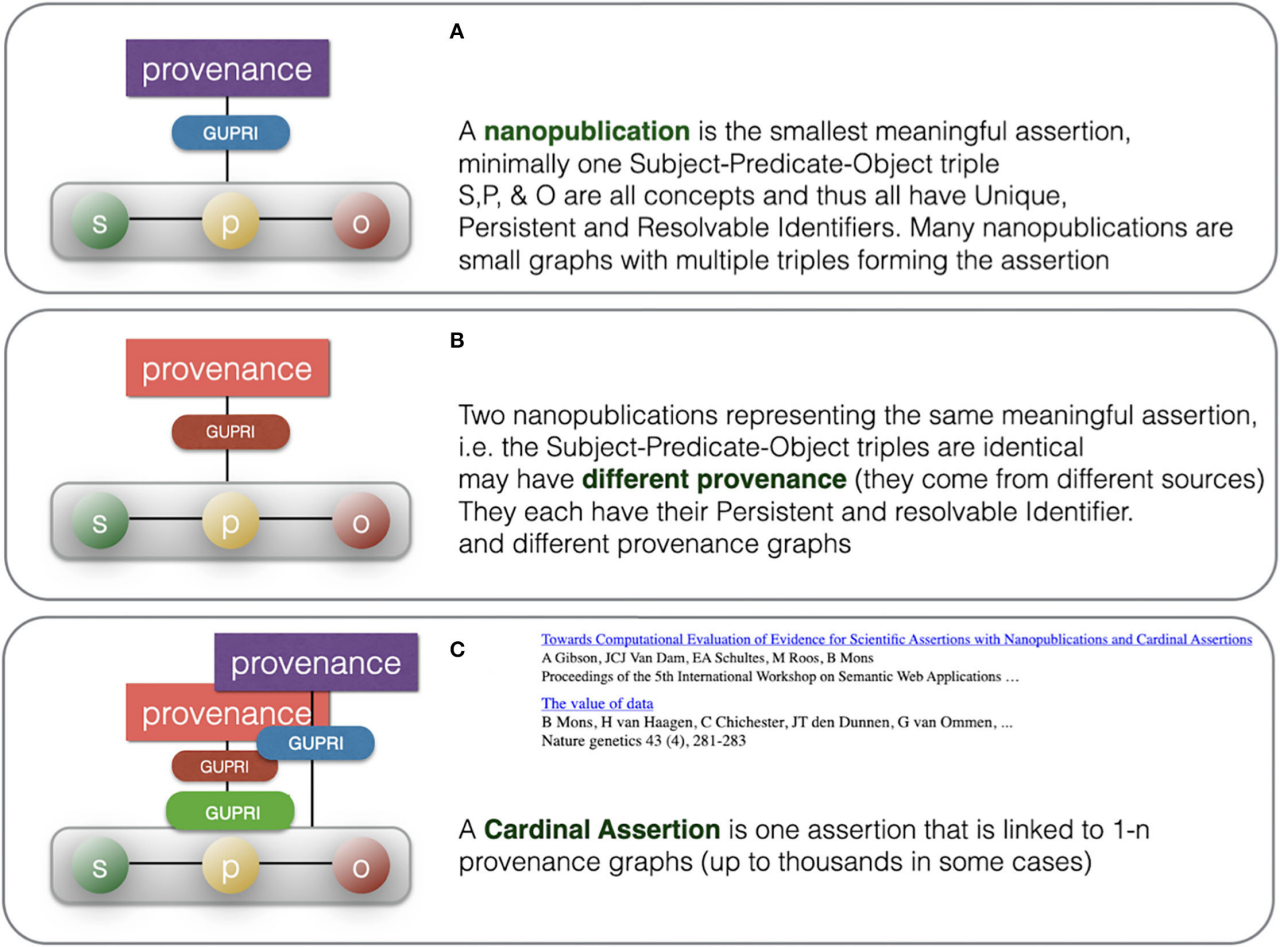
Summary of the consolidation from multiple nanopublications with identical assertions to once cardinal assertion with multiple provenance files, allowing detailed study of the different sources for this assertion. **(A)** depicts a typical nanopublication having an assertion (green-yellow-red) and provenance (purple) while **(B)** is a different nanopublication having the same assertion (green-yellow-red) but different provenance (red). **(C)** depicts how the single cardinal assertion (green-yellow-red) links to many independent nanopublications (multiple provenance). In computer reasoning, the use of the actual provenance files will be limited, although they can be used as a source for a numerical “evidence level” representing a subjective level of trustworthiness in a given context and for a given purpose. Metadata files that state contesting opinions and rendering the assertion less likely or even controversial can be used as well.

**Figure 9:**
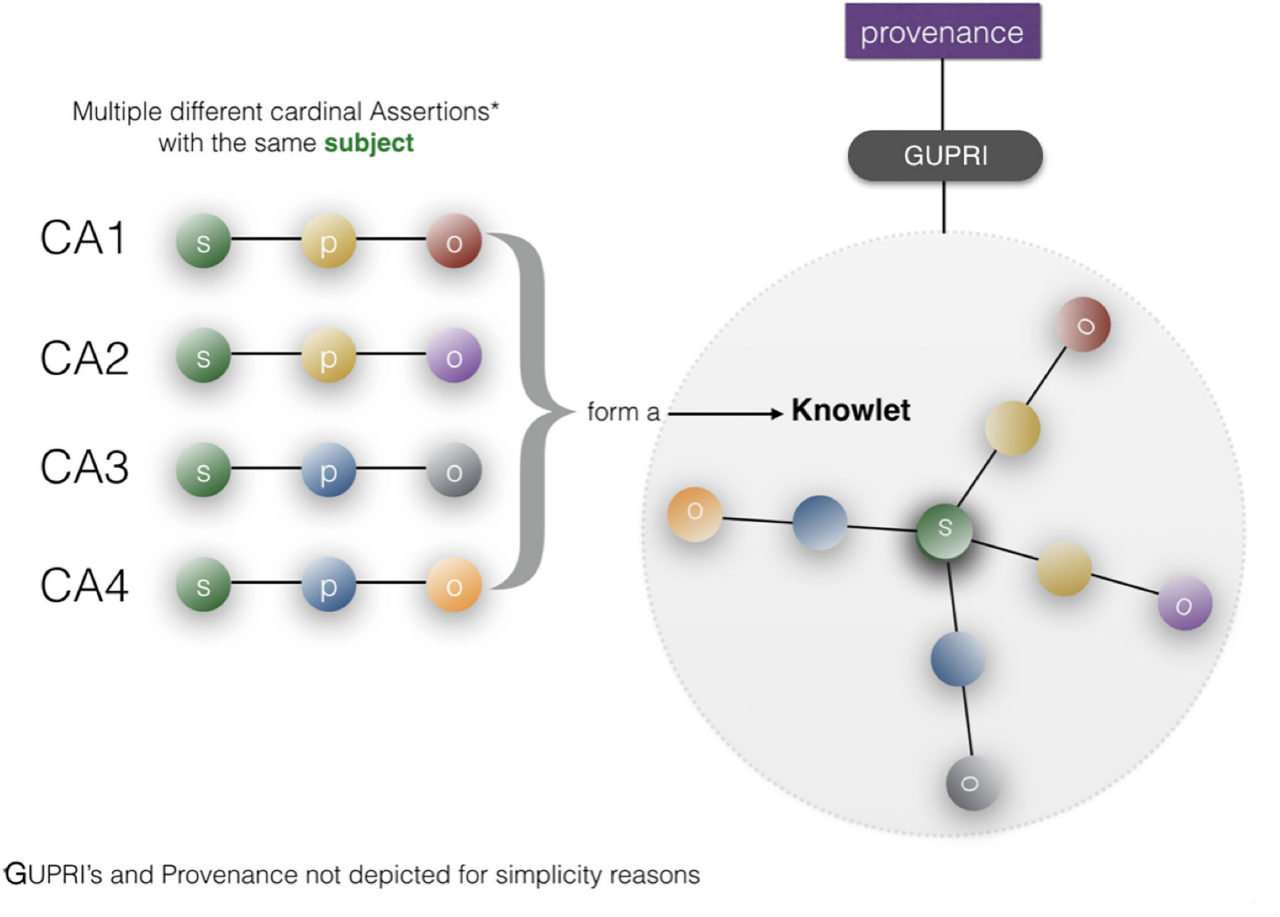
A Knowlet file is created from cardinal assertions with the same subject (GUPRI) [adapted from Mons (2019)]. A typical Knowlet will contain hundreds, thousands or even millions of FDOs (GUPRIs for all concepts, Cardinal Assertions, and custom references to external provenance files). By filtering the cardinal assertions in a Knowlet based on any type of recorded feature, such as predicates, object-semantic types, for instance “drug,” or time date stamps) we can create a “qua” (Masolo et al., 2005; Guizzardi, 2006) of any Knowlet for customized use.

Here, the FDO rule that metadata should be separated from the actual FDO they describe becomes critically important: each of the “supporting” identical nanopublications has one or more different metadata containers^11^ (themselves constructed as a proper FDO) associated with it, pointing to its GUPRI. The cardinal assertion will have links to all the metadata containers (enabling retrospective expansion to all nanopublications with their full provenance) but it can also be linked (for performance purposes) to a single metadata container (with its own FDO status and GUPRI) that contains all metadata files, and which can be “expanded *ad hoc*.” An example of a proprietary implementation of this principle, is the Dutch company Euretos^12^ where it is used in their AI Platform to be able to handle reasoning over vast amounts of triples effectively in near real time. Please note that all the below figures of nanopublications (Figure 6), cardinal assertions (Figures 7, 8) and Knowlets (Figure 9) are artist impressions, heavily simplified for human readability reasons, but they will all be constructed in real instances as FDOs and adapted to the finally agreed specifications of FDOs once these are formally published.

**Figure 10:**
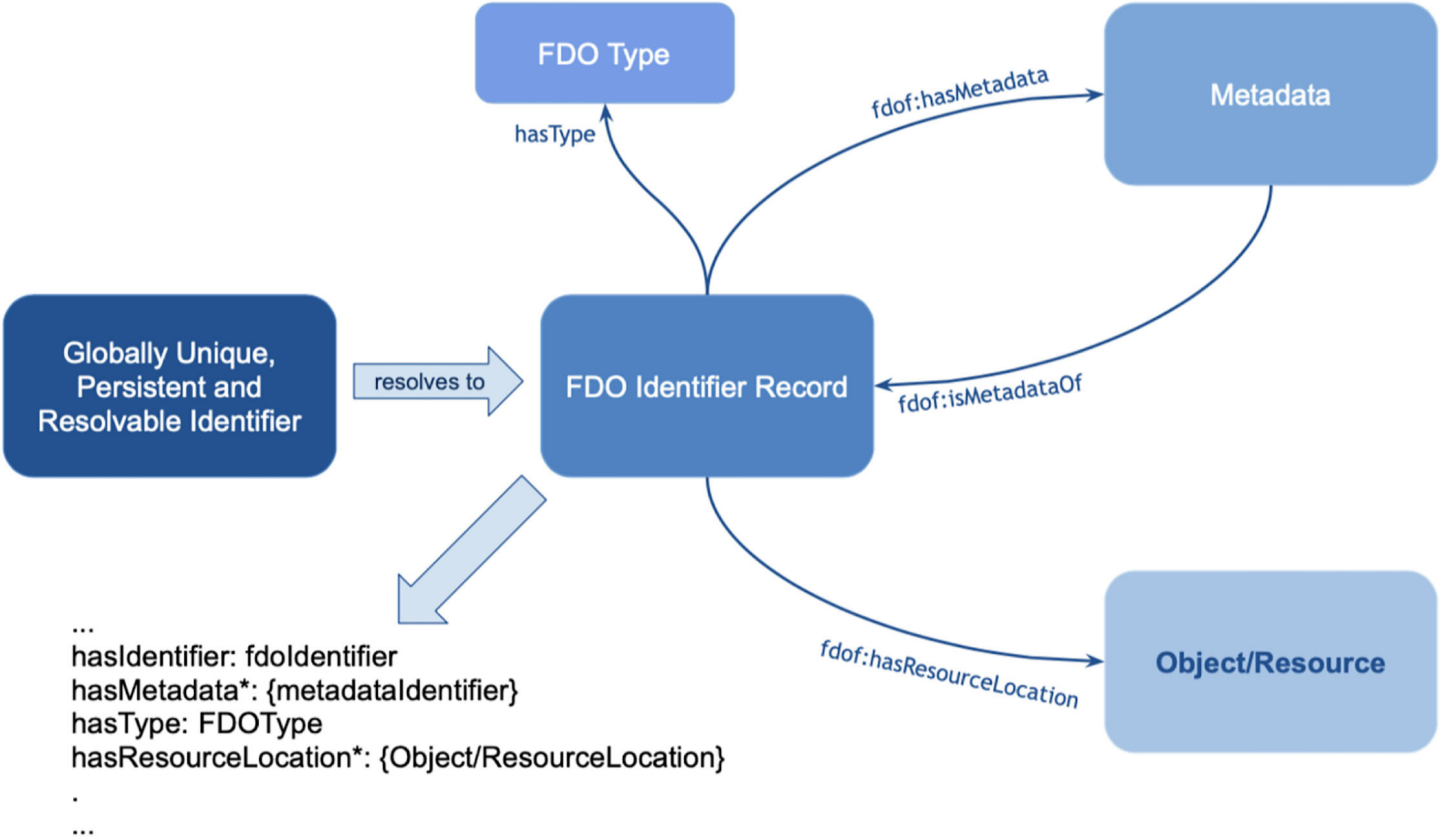
The basic anatomy of a FAIR Digital Object (FDO) (see text footnote^9^). The object (resource) in this case is the actual assertion (single triple or small graph), the FDO Identifier record is associated with a GUPRI that resolves uniquely to this nanopublication. The FDO type is “nanopublication,” which would inform machines about the generic technical actions possible with this type. The metadata (which are usually multiple files) as shown in Figure 5 are stored in a separate container and linked with the predicate < fdof:isMetadataOf> to the FDO identifier record (GUPRI).

In Mons (2019) the construction of a so-called Knowlet from cardinal assertion building blocks has been described in more detail: From the current (FDO) perspective (Figure 10), a Knowlet is a cluster of all cardinal assertions that share the same GUPRI as a subject. The resulting Knowlet is now entirely built from FDOs, and consequently forms a novel FDO itself, with its own type (Knowlet), its own GUPRI and its own metadata file(s) associated. The most complete Knowlet representing a concept thus represents all cardinal assertions that have been collected about its central subject (represented by its GUPRI). That GUPRI can refer to a physical object or resource (including a person), another abstract concept (a disease, a gene, etc.) or to another digital object (a nanopublication, a database, a workflow, etc.) or in this case of this article an FDT. In that sense, a Knowlet can also be seen as a form of ‘metadata' about the subject referenced by its central GUPRI.

## Fair Digital Twins May Have Effects on Machine Assisted Reasoning and Machine Learning

Once we approach the Knowlet as a particular form of a rich ***metadata file*** for all kinds of concepts (referred to by the central subject GUPRI in the Knowlet), we can start to see additional emerging features that can crucially support machine learning and address some of the inherent weaknesses of earlier fixed ontologies and static knowledge graphs, such as fixed relations between concepts (only). These emerging features of Knowlets have been described earlier in but are briefly revisited here in the novel perspective of FDTs as FDOs.

Here we discuss how Knowlets, as a format for FDTs, can serve in a knowledge recovery and discovery environment. First, we emphasize here once more that a Knowlet can “describe” any type of concept. For instance, a molecule, a data collection, a workflow, a cell, a tissue, an organoid, a disease, a person, a sample in a biobank or a collection, but also an individual “cow” or an entire city. As all composing elements of the Knowlets are cardinal assertions (FDOs) with full, but separated FAIR provenance, Knowlets can be “sliced and diced” in any way shape or form as required, to create customized *quas*, which are relevant subsets of Knowlets, or different views, or semantic lenses on the concept they represent. Thus, metadata (Knowlets) themselves can be the substrate for research, while the subjects they refer to (which could also be another, more elaborate data file) can be addressed as well, and in the case of the subject being a FDO itself it can be also addressed as substrates for machine assisted reasoning, analytics, or learning. This allows for machine actionable (FAIR) metadata to render the data files they refer to, for instance a CSV, VCF, or DICOM file to become machine actionable, without being intrinsically FAIR. in fact, the Knowlet, as an FDO, will serve the Findability, the Accessibility (including license). In addition, the Knowlet can contain machine readable instructions about “what to expect” in terms of data files in the next “container.” There could even be an assertion in the Knowlet regarding the level of FAIRness of the data set it refers to. So, machines can ultimately also be instructed that the data in the next container are not machine actionable and need to be processed first. This could include free text to be analyzed by text mining technologies, or even structured data such as a spreadsheet without proper references for the columns and the rows and/or with ambiguous terms in the cells.

**Figure 11:**
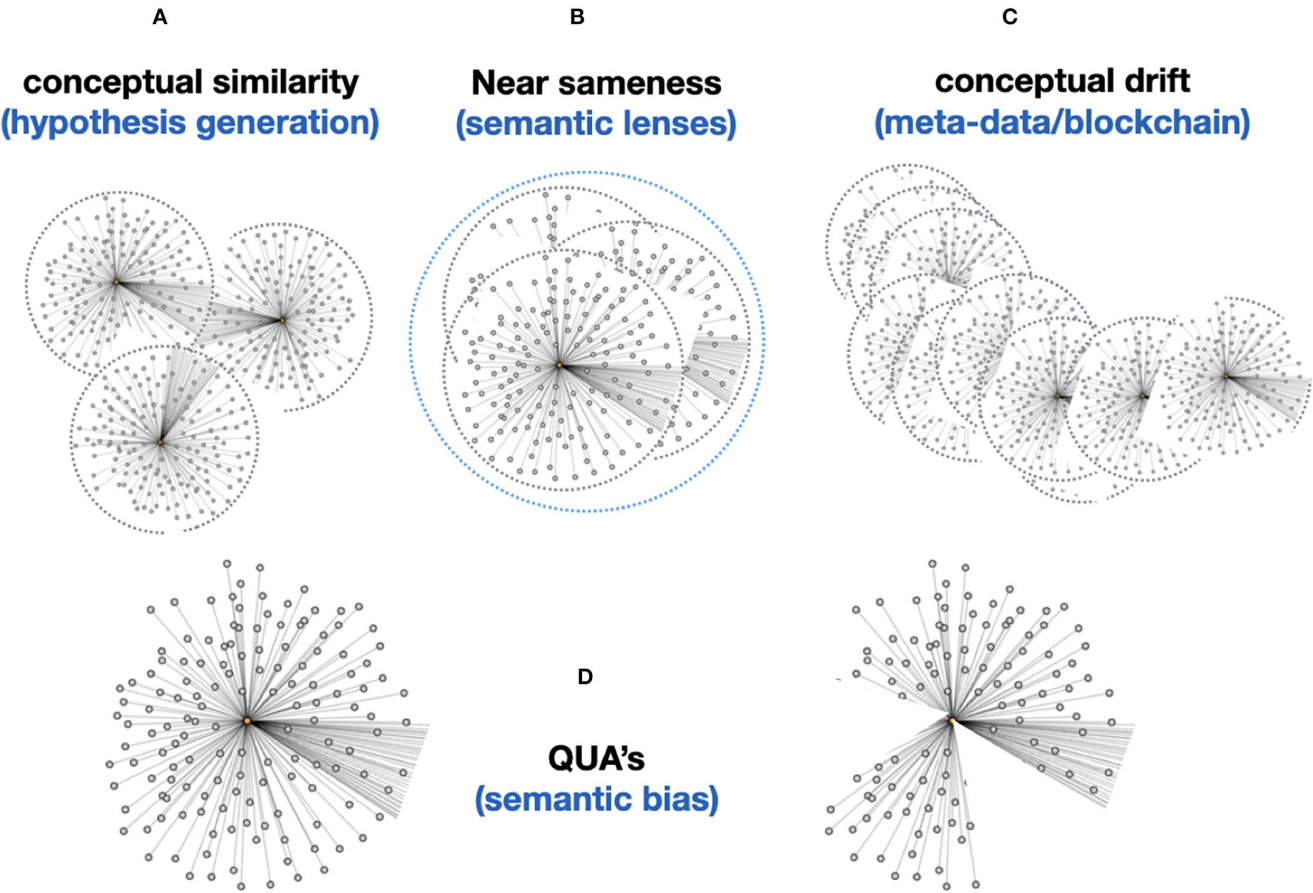
**(A)** The “conceptual similarity” of two Knowlets (FDTs) can be simply calculated based on existing vector matching approaches. This allows basic hypothesis generation (association between two Knowlets of concepts that have so far been never directly associated). **(B)** Now that machines are able, coming closer to human perception, to see the subject of the Knowlet in a much broader conceptual context (associating all objects and predicates in the Knowlet with the subject), computers are able to much better deal with “near sameness” and subtle semantic differences, without explicitly being instructed via fixed predicates. In static knowledge graphs or ontologies, we cannot simply define a predicate like “nearly identical as,” because this is an intrinsically undefined and thus elusive concept to machines. However, if in the picture **(B)** these three Knowlets for instance represent the homologous gene in *H. sapiens, M. musculus, and R. norvegicus* (man, mouse, rat), the vast majority of the millions of concepts (FDOs) in the three Knowlets may be identical. That means that the machine “knows” that these three concepts are distinct -because they have different central subject GUPRIs as well as overall container GUPRIs- but are “nearly similar” and it “knows” the -relative- extent to which they are similar. Later we will see that their quas (filtered from all concepts related to species and chromosomal location) will be identical. **(C)** Importantly, although single nanopublications (snapshots of an assertion made at a given time) and the content of cardinal assertions should be immutable, and protected by for instance Trusty URIs (Kuhn and Dumontier, 2014), the Knowlet representing a concept is principally mutable and drifting. For instance, if the Knowlet refers to a gene, any new variant found in an instance of the gene detected by sequencing research will add a new assertion to the overall Knowlet of that gene as a mental construct. This will effectively create a new version of the Knowlet (FDO), with a new GUPRI, which in most cases will be 99.999% identical to its immediate predecessor, and secondly can contain the assertion [new GUPRI] previously known as [predecessor GUPRI]. Effectively creating a block-chain type sequence which enables backward recovery of earlier versions of the Knowlet, effectively supporting versioning (for instance of a workflow) or semantic drift detection (of a concept over time or by geographical region/culture). **(D)** Finally, each Knowlet can be “filtered” on any feature that is supported by the internal or externally associated content. For instance, on time date stamps of individual cardinal assertions in the Knowlet, on predicate type, or on the semantic type of the objects in the cardinal assertions. Coming back to **B** (near sameness): assuming that a very conserved gene/protein like actin would be identical in man, mouse, and rat, the three Knowlets of these three distinct concepts would at least differ on two cardinal assertions, determining the species and the chromosomal location of the gene. The Knowlets will be nearly similar even without filtering, but it will be a relatively straightforward machine-instruction to “ignore species and chromosomal location” and these Knowlet quas will now be actually 100% identical and treated in any graph-reasoning as “one and the same concept” until there is a need to separate them again. Finally, the qua approach might also reduce or even eliminate the need for new GUPRIs for each version of a developing FDT. In fact, older versions can be simply recovered by filtering on all cardinal assertions that were added before a given time.

Moreover, Knowlets that represent concepts in a knowledge space (for instance drugs and diseases) can now be dynamically used to determine the level of “sameness” in terms of relevant overlaps in their respective concepts (Figure 11A). We have already shown the value of this approach in the prediction of novel gene-disease and protein-protein interactions (Jelier et al., 2005; van Haagen et al., 2009, 2011, 2013; Hettne et al., 2016) but in principle this approach can be replicated for any pair of semantic types (for instance similarity between two cities. When two Knowlets are extremely similar, but the GUPRIs of their central concepts differ, machines now have an independent way of inferring the extremely similar conceptual positioning in knowledge space of these two independent Knowlets. As explained in Figure 11B this can also be refined by filtering Knowlets on certain semantic types or relations that are deemed irrelevant for certain studies or purposes. For instance, the *quas* of the highly conserved protein Actin from different species (where the gene is on a different chromosome and the species is explicitly different, can be rendered “identical” by determining the semantic types “species” and “chromosomal location” as irrelevant (for this purpose). Obviously, as stated before, Knowlets will grow over time when new insights about the central concept emerge. The position of the Knowlet in conceptual space will change in that case. Here we define that as conceptual drift (Figure 11C). Since the *qua* concept also contains the filtering on time date stamp of each of the cardinal assertions in the Knowlet, it is always possible to reconstruct the Knowlet as it was at a given point in the past. This avoids the need for the permanent storage of a long series of FDTs over time. There is an intrinsic way to track the delta of Knowlets because of the implementation of Trusty URIs (Kuhn and Dumontier, 2014) as GUPRIs for the Knowlet as a whole (see Figure 9) that will change automatically when the hash-code of the content of the FDO changes. We may also consider the implementation of genuine block chain technologies with a scalability and low footprint such as Cardano^13^ but we will only use these energy-consuming technologies when they appear necessary for tracking and tracing and for smart contracts.

## Discussion and Implementation Considerations

### Knowlets in a Concept Space of “Types” or “Mental Constructs”

Consider the enormously large concept space of health-related life sciences. The concepts we publish about in Medline (> 40 million abstracts) are in most cases ‘types' (genes, proteins, metabolites, chemicals, cell types, tissues, organs) rather than physical instances of types (TP53 Gene, Tumor Protein P53, lactic acid, sodium chloride, satellite glial cell, simple glandular columnar epithelium, heart—respectively). We will address real world observations on particular individuals later in this discussion.

The formatting of FDTs of these mental constructs (types) as Knowlets, being as close to current views on FDOs as possible, leads to a number of very interesting emerging features of FDTs that may give them great potential for the transition to real “machine intelligence.” First of all, the number of concepts that we use in reasoning is limited. For the entire life sciences field an “educated estimate” of all nanopublications (in fact assertions, regardless of their format) that have been created over the 30 + years in Medline would be in the order of 10^14^. Obviously, the imaginary collective triple store of all nanopublications would contain an order of magnitude more triples, as each nanopublication is linked to elaborate provenance (including for instance all authors of the article it comes from). Although this provenance can be stored separately from the actual assertions, as practiced also in the Euretos environment, a graph containing all 10^14^ nanopublications would still be a very significant, and as yet unworkable graph.

The first of 3 orders of magnitude reduction can be achieved by only storing cardinal assertions (~10^11^) and, indeed, separating these from the large metadata files. Regardless of the fact that we will never be able to rescue all the 10^14^ nanopublications from legacy narrative resources of the past, we know for a fact that, once clustered by subject, we only have <*1 million* (10^6^) *Knowlets* with any serious content in the entire biomedical life science database ecosystem.

In other words, the multidimensional conceptual space, now captured as a “fluid graph” of free moving Knowlets (as opposed to a static Knowledge graph in ontological fixed format) does contain only 1 FDTs, and their multi-dimensional conceptual overlap calculations are relatively trivial, as is the storage needed for this 1 million by 1 million matrix. In addition, the number of semantic types in this concept space only amounts to a large, but manageable number of predicates possible between each given pair of subject-object semantic types is also limited. Effectively, we can re-engineer a “conceptual model” of the entire life sciences concept space based on what has been “actually asserted” so far that forms an incredibly rich and powerful substrate for research, as proven by the industrial application of Euretos.

## Real World Observations and FDTs of People and Objects

**Figure 12:**
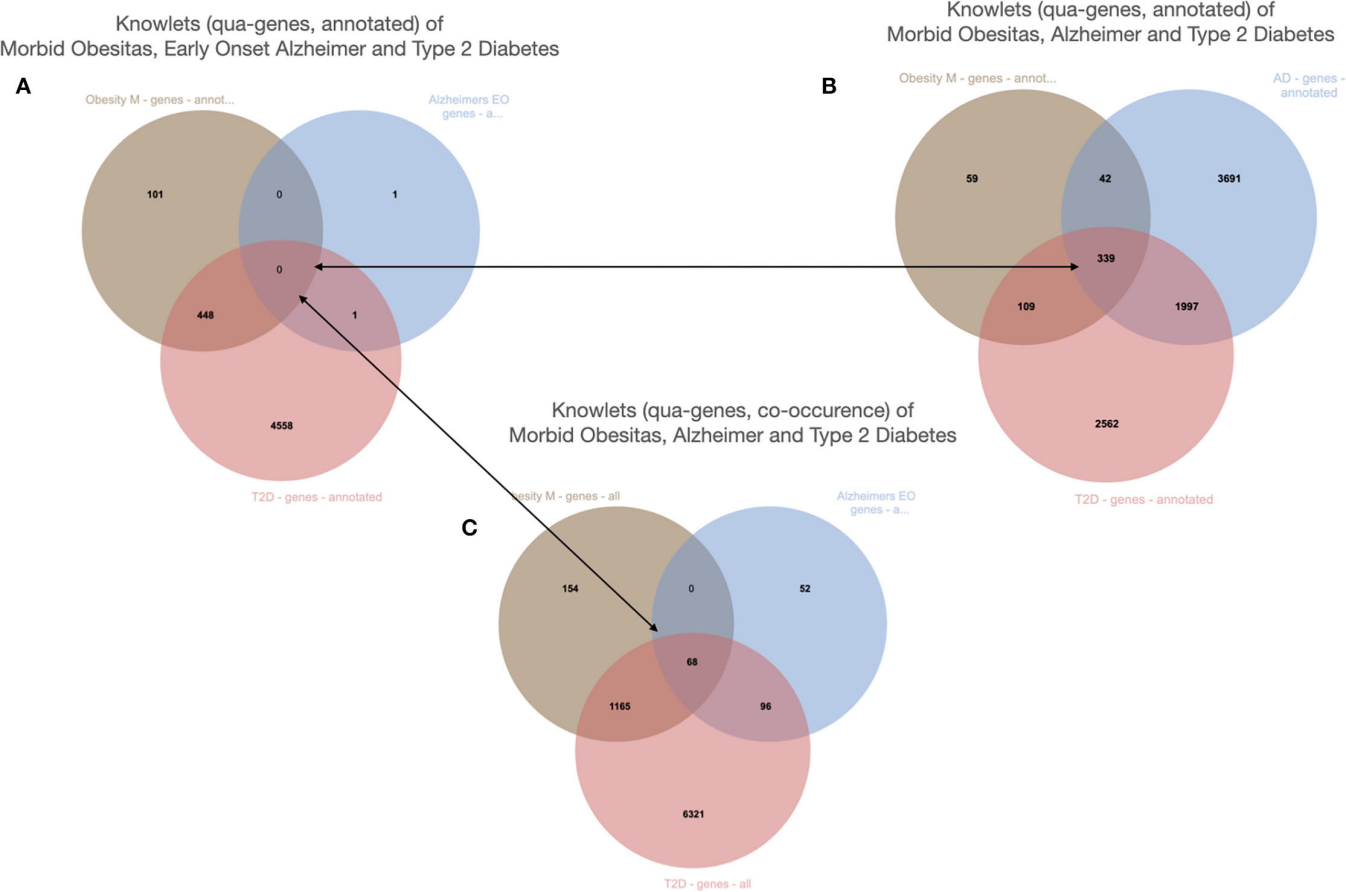
The Knowlets (strongly filtered quas) of three diseases and their gene overlap (Euretos interface). Panel 1 is filters on the semantic type “gene” (object) and for curated annotations only (predicate). No overlap in genes is detected. **(B)** The Knowlet is expanded to the broader concept of Alzheimer's disease. **(C)** The filter of **(A)** is the same but now all literature co-occurrences (not -yet- annotated and some potentially spurious) are included.

Real world observations on physical entities (people, blood samples, machines), which are ontologically spoken instances of a type, can also be recorded in machine readable format and made FAIR through rich, machine actionable metadata. We effectively create FDTs of real-world entities. It is important for research to separate FDTs representing real word observations on physical entities, for instance all genomics, metabolomics, proteomics, and exposome data observed for a particular person, from “established knowledge” graphs, such as for instance the Euretos Graph that we explore for open-ended research. This allows for accidental and anecdotal real-world observations to be “rationalized” in established knowledge graphs (exploring the relationships between the types of the instances we observe (see for instance Figure 12). Reciprocally, when a hypothesis emerges from established knowledge analysis, it becomes feasible to study whether the hypothesized correlations actually emerge from real world data.

Having first separated, and then in a controlled manner, re-combine established knowledge graphs with the real-world observations, creating a fundamentally new approach to biomedical research, and indeed, science itself. Smart machine analytics and learning algorithms can reason over these fluid graphs in real time and in the example of adjusting the setting of a *qua* “filter” will shift the position of the affected Knowlets, in the conceptual space leading to new perspectives on the same “reality.” As we can (and will) also record the source of each cardinal assertion, also when it comes from a thesaurus or an ontology, we can “view” the concept “water” for instance limited to the “semantic lens” of the Sea Data Cloud consortium (only allowing triples from their ontologies of choice). Not unlike the metaphor of Indra's Net, the nodes and edges of the knowledge graph provides to any researcher a personal point of view of the entire knowledge space, conditioned by their own knowledge, experience and scientific intuitions, while at the same time FDT representations of the graph, allow these private views to be documented and communicated to others with precision.

In a sequential article we will describe the developing FAIR orchestration architecture to deal effectively with distributed analytics over FAIR data and in particular FDTs. Although the concept of a digital twin is getting some traction in the life sciences field (Hernandez-Boussard et al., 2021) we would like to emphasize that digital twins need to be FAIR in order to be “AI ready” (i.e., machine actionable and interpretable) to be optimally repurposable. To support this argument, we give here a more current example that allows an insight in how cardinal assertions and Knowlets can already be used with existing infrastructure (www.euretos.com) for rapid rationalization of real-world observations using curated established knowledge.

The example is from unpublished and potentially sensitive information and therefore we will not go into the biology itself and any individual genes that were studied in this case in any depth, but for the sake of demonstration, we describe the anecdotal observation leading to a hypothesis that there could be a correlation between diabetes type 2, morbid obesity, and early onset Alzheimer's disease (Figure 11). The figure demonstrates a number of things. First, we need to deal with synonyms, for example “diabetes type 2,” “T2D” (type 2 diabetes) and (see Figure 12) “diabetes mellitus, insulin dependent.” Symbols, including both natural language terms and identifiers referring to this disease, as a “mental construct” should, although referred to by many synonyms and multiple “uris” in multiple thesauri, always resolve to the intended defined meaning. The other aspect the pictures demonstrate is the option of “*quas*” (subsets of the full FDT selected for a custom purpose). In this example, for simplicity's sake, only the semantic type “gene” was selected and the Knowlets of the concepts Morbid Obesity (brown), Early Onset Alzheimer's disease (blue) and Type 2 Diabetes (red) were further filtered for “annotated relations” from curated databases *only*. In panel A, the three *quas* filtered with these settings, no genes are found that are formally annotated with all three diseases, which is a proxy for the absence of formal, explicit, and curated knowledge about a possible genetic component that could support a mechanistic rationale for the perceived correlation between early onset Alzheimer's disease with a combination of the other two in real world setting. However, when the *qua* of Alzheimer's disease in a broader sense is used instead (panel B), there appear to be 339 Alzheimer's associated genes that have curated co-annotations with morbid obesity *and* T2D in curated databases. This might indicate genes that are candidates for study in this context. When the *qua*-filter for “curated relationships only” is removed, the first combination used in panel A yields 68 genes that have a non-specified co-occurrence in the same article with all three diseases (Panel C). The combination of these three different Knowlet (*qua*) based panels would now quickly focus the attention of researchers in the most likely genes to explore further.

**Figure 13:**
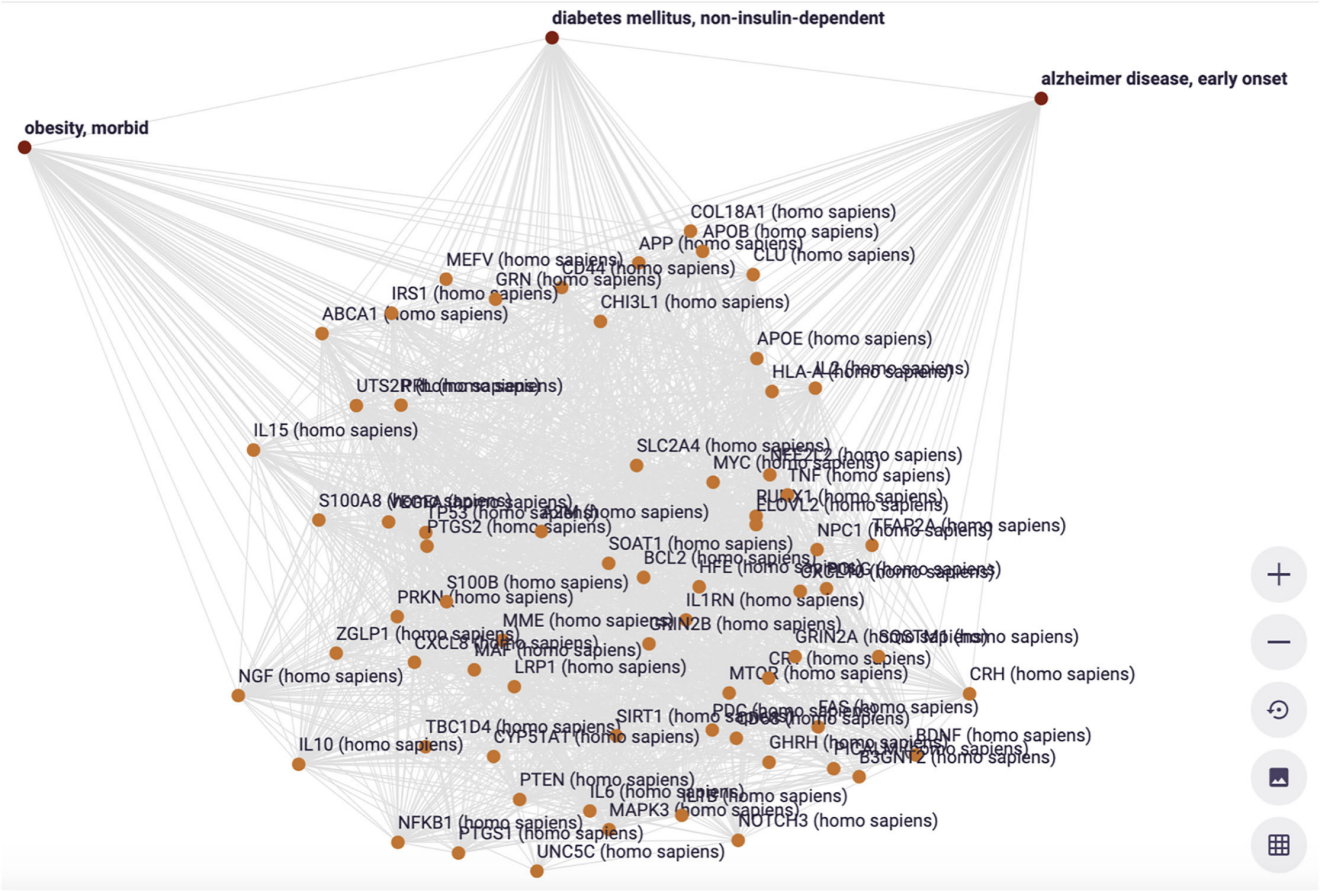
The visualization of the interconnections between the 68 genes associated with three diseases (filter setting is Figure 11C) (Euretos interface). Each line in this graph is representing a cardinal assertion (as in Figure 8) and all provenance (type of relation = predicate), time date stamp and (mostly multiple) sources of that cardinal assertion can be explored (effectively showing all nanopublications supporting the assertion (as in Figure 6).

In Figure 13, a visualization of a subset of overlapping genes is shown, including their curated interrelationships. This is the start of a deeper exploration of genes that seem highly interconnected as well as associated with all three disease manifestations.

More recently we have applied the combination of real world observations on COVID-19 patients with the established knowledge graph and could rationalize the mechanism of action of dexamethasone on the overproduction of prostaglandin e2 in severe patients (Mons et al., 2020). These rationalizations where further explored using organ-on-a-chip technology and perfusion of patient plasma through intact lung tissue organoids demonstrated an increase of the prostaglandin levels beyond the original concentration, indicating the presence of inducing precursors of prostaglandin production. Meanwhile, the effectiveness and mechanistic explanation of dexamethasone treatment is well-established, but we have also rationalized several other observations on the effect of drugs and monoclonal antibodies in COVID-19 patients, and we are starting to apply the same approach to long-COVID, which is a complex and multifactorial syndrome partly caused by SARS-CoV-2 viral remnants, but also in some cases by co-infection flare up of latent viruses in the patient.

## Conclusion

We have reviewed the different developmental phases that finally led to the comprehensive conceptualization of a FAIR Digital Twin representing real world objects as well as “types” and mental constructs such as the concepts of particular genes and diseases. We also described the high level construction process of FDTs from nanopublications to cardinal assertions to Knowlets and how we anticipate to adapt these (if needed) to follow the exact formatting needed to make the vanilla FAIR Digital Objects as soon as the full specifications of this new type of machine actionable units of information will be approved by the global expert community. We use the proxies we have available today already for effective machine assisted knowledge discovery for several years and we are in a process of building a supporting infrastructure that will be adopted throughout Dutch health research as well as increasingly for AI applications in other domains. This article serves as a “credo” for a collaboration among the institutions to which the authors are affiliated. Toward building this collaboration on firm foundations, we aim to publish a technical article describing the computer science and information architectures behind the processes needed to increasingly automate the knowledge discovery processes with Knowlets and FDTs that are today performed mostly by human labor data munging. All code will be open source and published in open access environments.

## Data Availability Statement

The original contributions presented in the study are included in the article/supplementary material, further inquiries can be directed to the corresponding authors.

## Author Contributions

ES and BM conceived of the generic concept of FDTs. LB conceived of the adapted FDO specs. MR reviewed the manuscript. GG and LB added the basic concepts of conceptual modeling and Qua entities. AB created the example analysis. TH reviewed the manuscript. All authors contributed to the article and approved the submitted version.

## Funding

Part of the research leading to this design of FDTs was funded by the Leiden Center of Computational Oncology, an internal grant of the Leiden University Medical Center and NeXON (Next-Generation Ontology-Driven Conceptual Modeling) financed by UNIBZ-CRC. The design of novel nanopublications was partially funded under the project “Trusted World of Corona”, TKI-LSH Health~Holland grant number LSHM20070.

## Conflict of Interest

ES and BM were employed by GO FAIR Foundation. AB was employed by Euretos. The remaining authors declare that the research was conducted in the absence of any commercial or financial relationships that could be construed as a potential conflict of interest.

## Publisher's Note

All claims expressed in this article are solely those of the authors and do not necessarily represent those of their affiliated organizations, or those of the publisher, the editors and the reviewers. Any product that may be evaluated in this article, or claim that may be made by its manufacturer, is not guaranteed or endorsed by the publisher.

## Footnotes

^1^FAIR Digital Objects Forum (2022). Available online at: www.fairdo.org (accessed: February 23, 2022).

^2^Health-RI. (2022). Available online at: https://www.health-ri.nl/ (Accessed February 23, 2022).

^3^EOSC Association (2022). Available online at: https://eosc.eu/ (accessed February 23, 2022).

^4^Health~Holland (2021). *Citizen Centred and Controlled COVID-19 Data for Reuse*. Available online at: https://www.health-holland.com/project/2021/2021/citizen-centred-and-controlled-covid-19-data-reuse (accessed February 23, 2022).

^5^Polymorphic Encryption and Pseudonymisation (PEP) (2017). Radboud University. Available online at: https://www.cs.ru.nl/B.Jacobs/TALKS/pep-overview.pdf

^6^FAIR Digital Object Forum (2022). *FDO-TSIG Technical Specification and Implementation Group*. Available online at: https://fairdo.org/wg/fdo-tsig/ (accessed: February 23, 2022).

^7^GO FAIR Foundation (2022). *GFF Interpretations and Implementation Considerations for Each FAIR Guiding Principle*. Available online at: https://osf.io/vzhda/ (accessed February 24, 2022).

^8^W3C Semantic Web (2021). RDF. Available online at: https://www.w3.org/RDF/ (accessed february 23, 2022).

^9^Nanopub.org (2013). Available online at: https://nanopub.org/nschema (accessed February 23, 2022).

^10^Nanopublications. *FDO|Fair Digital Object* (2022). Available online at: https://np.petapico.org/RABv2ScOD0VlRskTF3WaQhc8gtIy0_MtEVzDKPitRPaRo#FDO (accessed February 23, 2022).

^11^FAIR Digital Object Framework (2021). *Documentation*. Available online at: https://fairdigitalobjectframework.org/ (accessed February 23, 2022).

^12^Euretos (2022). Available online at: https://www.euretos.com/ (accessed February 23, 2022).

^13^Cardano (2022). Available online at: https://cardano.org (accessed February 23, 2022).
